# Genomic Insight into Differentiation and Selection Sweeps in the Improvement of Upland Cotton

**DOI:** 10.3390/plants9060711

**Published:** 2020-06-03

**Authors:** Mian Faisal Nazir, Yinhua Jia, Haris Ahmed, Shoupu He, Muhammad Shahid Iqbal, Zareen Sarfraz, Mushtaque Ali, Chenfan Feng, Irum Raza, Gaofei Sun, Zhaoe Pan, Xiongming Du

**Affiliations:** 1 Institute of Cotton Research, Chinese Academy of Agricultural Sciences, State Key Laboratory of Cotton Biology, Anyang 455000, China; mfn121@hotmail.com (M.F.N.); jiyayinhua@caas.cn (Y.J.); hafizahmed25@hotmail.com (H.A.); heshoupu@caas.cn (S.H.); shahidkooria@gmail.com (M.S.I.); zskpbg@hotmail.com (Z.S.); mushtaqjan6@gmail.com (M.A.); irumkhattak@gmail.com (I.R.); 20160380@ayit.edu.cn (G.S.); panzhaoe@caas.cn (Z.P.); 2 Research Base, State Key Laboratory of Cotton Biology, Zhengzhou University, Zhengzhou 450001, China; 3 Ayub Agriculture Research Institute, Cotton Research Institute, Multan 66000, Pakistan; 4 Department of Electronics and Information Engineering, Sichuan University, Chengdu 610000, China; Chenfanfeng1983@gmail.com; 5 School of Agricultural Sciences, Zhengzhou University, Zhengzhou 450001, China

**Keywords:** upland cotton, phylogeny, domestication, selection sweeps

## Abstract

Upland cotton is the most economically important fibre crop. The human-mediated selection has resulted in modern upland cultivars with higher yield and better fibre quality. However, changes in genome structure resulted from human-mediated selection are poorly understood. Comparative population genomics offers us tools to dissect the genetic history of domestication and helps to understand the genome-wide effects of human-mediated selection. Hereby, we report a comprehensive assessment of *Gossypium hirsutum* landraces, obsolete cultivars and modern cultivars based on high throughput genome-wide sequencing of the core set of genotypes. As a result of the genome-wide scan, we identified 93 differential regions and 311 selection sweeps associated with domestication and improvement. Furthermore, we performed genome-wide association studies to identify traits associated with the differential regions and selection sweeps. Our study provides a genetic basis to understand the domestication process in Chinese cotton cultivars. It also provides a comprehensive insight into changes in genome structure due to selection and improvement during the last century. We also identified multiple genome-wide associations (GWAS associations) for fibre yield, quality and other morphological characteristics.

## 1. Introduction

Cotton (*Gossypium hirsutum*) is a major source of fibre for the textile industry, especially tetraploid cotton which covers 95% of the worldwide cotton production. Selection has been carried out in cotton to improve production and adaptation to the local environment, reduced growth period, and its defence against biotic and abiotic factors. Due to continuous selection pressure, the cotton crop is facing a narrowed genetic base in terms of diversity [1,2,3,4]. Therefore, insight into the genomic structure and changes occurring in genomic structure due to continuous selection and improvement can yield interesting information resulting in a better understanding of the process of domestication and improvement. 

Cotton has been grown in China for centuries. However, the introduction of upland cotton cultivars throughout the world has changed the production scenario of the cotton crop worldwide since upland cotton is occupying most of the production area of cotton. Prior to the introduction of upland cotton in China, a diploid species *Gossypium arboreum* was mainly grown in China. The earliest reported evidence suggested that the first introduction of tetraploid cotton into China was during the French colonist era [5,6]. The Second introduction of tetraploid cotton into China was at the beginning of the 20th century when upland cotton was systematically introduced into China. During these years, cotton was mainly distributed in the Yangtze River region and the Yellow River region in China [7].

In China, cotton has been produced in three main regions; Yellow River Valley (YeRV): Hebei, Shandong and Henan, northwestern China: Xinjiang province, The Yangtze River Valley (YaRV): Hubei, Hunan, Jiangsu, Anhui [8]. During recent years, cotton production has been mainly shifted to Xinjiang province. Prior to the introduction and implementation of Seed Law (SL) and Plant Variety Protection Act (PVPA) in China [8], most of the southwest varieties (introduced in early 20th century from the United States of America in the Yangtze River region and Yellow River region in China [7]) were maintained by farmers over the years. From this diverse southern gene pool, which is not only from different ecosystems but also has lower human intervention, useful information can be excavated to study the diversity of cotton crop in China through the evaluation of current Chinese varieties and their genetic background with reference to southern varieties and landraces from Central America. 

Improvements in genomic studies and resequencing technologies have established the tools to dissect the genetic basis of elite cultivars. Different techniques have been used to understand genetic diversity in upland cotton [2,9,10,11,12,13,14], i.e., pedigree breeding, morphological and biochemical markers, and molecular markers. In recent years, genome-wide association studies (GWAS) have proven to be a remarkable tool to dissect genetic diversity among cultivars to understand the genetic mechanism behind diseases and associations of putative candidate genes for morphological traits. GWAS have been widely implemented in maize, rice, Arabidopsis and legumes. In addition, single nucleotide polymorphism (SNP) genotyping techniques, third-generation sequencing has facilitated GWAS to provide better association results for morphological traits with their genetic background. In cotton, GWAS have been used to dissect genetic mechanisms underlying fibre quality traits [15,16,17,18], diseases [19] and verticillium wilt [20,21]. However, despite all the technological breakthroughs in genomics, contribution towards finding new genetic sources in crop plants has been limited.

Though multiple studies have been conducted to understand the genomic basis of domestication in different crops [22,23,24], there are very few studies addressing the domestication of the cotton crop with reference to high-density genomic data. Our study aims to provide a better insight into the changes in genomic structure due to human-mediated selection and improvement. It is worth mentioning that three distinct groups of genotypes viz. (i) Modern cultivars (mainly cultivated in YeRV and Xingjiang province), (ii) Obsolete cultivars previously grown in South China for more than 50 years without management, (iii) other identified *Gossypium hirsutum* landraces collected from North America, used in this study are the representation of the breeding history of cotton crop in China over the decades since the introduction of upland cotton in China. This study will provide insight into the differences between the genetic profile of current varieties, accessions collected from southwest China and geographical landraces of *G. hirsutum*, which could be further utilised in breeding to expand the narrowed genetic base of upland cotton. 

## 2. Results

### 2.1. Population Classification and Structure Variations

**Figure 1:**
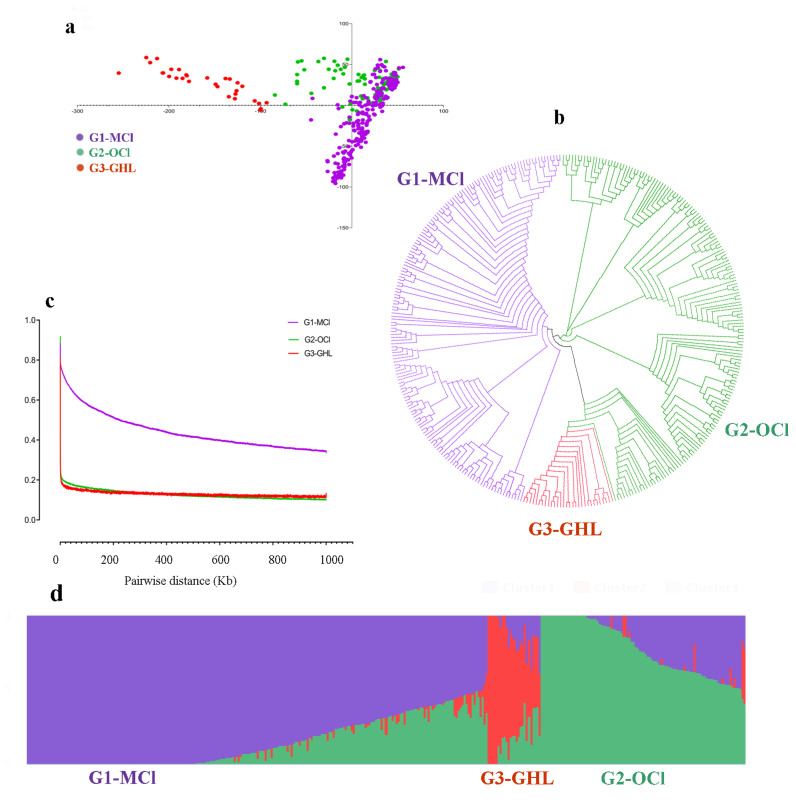
Population stratification. (**a**) Principal component analysis (PCA) plot of the first two PCAs, i.e., PCA1 (21.898%) and PCA2 (2.988%), for all accessions. Dot colour scheme is as G1-MCl = Modern cultivars G2-OCl = Obsolete Cultivars collected from south china, G3-GHL = Geographical landraces of *G. hirsutum,* (**b**) Phylogenetic tree constructed using whole-genome data, distributing genotypes into three clades as per original classification, (**c**) Pairwise linkage disequilibrium (LD) decay in each group, (**d**) Structure results for k-3.

We exploited the genetic relationship among all accessions using principle component analysis (PCA) and performed phylogenetic analysis to construct a phylogenetic tree using 4,329,838 single nucleotide polymorphisms (SNPs). The inference drawn from phylogenetic analysis, structure and PCA supported the classification into three groups (Figure 1). Group 1 comprised modern cultivars (MCl), while group 2 and group 3 were biased towards obsolete cultivars from South China (OCl) and geographical landraces of *G. hirsutum* (GHL)*,* respectively. Some accessions showed admixed ancestry suggesting the presence of introgression or gene flow during the breeding process (Supplementary Table S2). Phylogenetic tree complimented breeding history of *G. hirsutum* in China. In addition, linkage disequilibrium (LD) decay was measured as the physical distance on the chromosome (kb) when LD decreased to half of its maximum value. Linkage disequilibrium is critical in understanding and determining the location of causal loci through GWAS [25]. Furthermore, patterns of LD decay between different populations can present with the information regarding selective sweeps and selective pressure [26]. LD decay was observed at 357 kb (physical distance between SNPs) for MCl, while it was lower at 0.2 kb and 0.05 kb for OCl and GHL, respectively. These results indicated the linkage decay in the subpopulations of obsolete cultivars (OCl) and landraces of *G. hirsutum* (GHL) declined dramatically compared with that in modern cultivar (MCl) populations, which are in agreement with previously published statistics [27]. Furthermore, the extent of LD decay was higher in the cultivated group than in obsolete accessions and landraces, signifying the potential role of selection pressure, geneflow and nonrandom mating in shaping modern cultivars. 

### 2.2. Differentiation and Selection Signals between MCl and OCl Group

Modern upland cultivars have been developed from limited resources [18] and are spread worldwide in cotton-growing countries. Obsolete cultivars collected from Southwest China refer to the first systematic introduction of upland cotton in china. This distinct gene-pool, adapted to the local ecosystem, was mainly maintained by farmers without any organised breeding techniques. At the beginning of the 21st century, modern upland cultivars were systematically introduced worldwide, including China. As a result of rigorous selection, these cultivars referred to high yield and good quality. To understand this selection procedure and changes in genome structure due to continuous selection, we identified selection sweeps regions by comparing the genetic background of OCl, MCl and GHL. Our results pointed out differential selection patterns between different sub-populations, i.e., from landraces to OCl and from OCl to MCl. 

**Figure 2:**
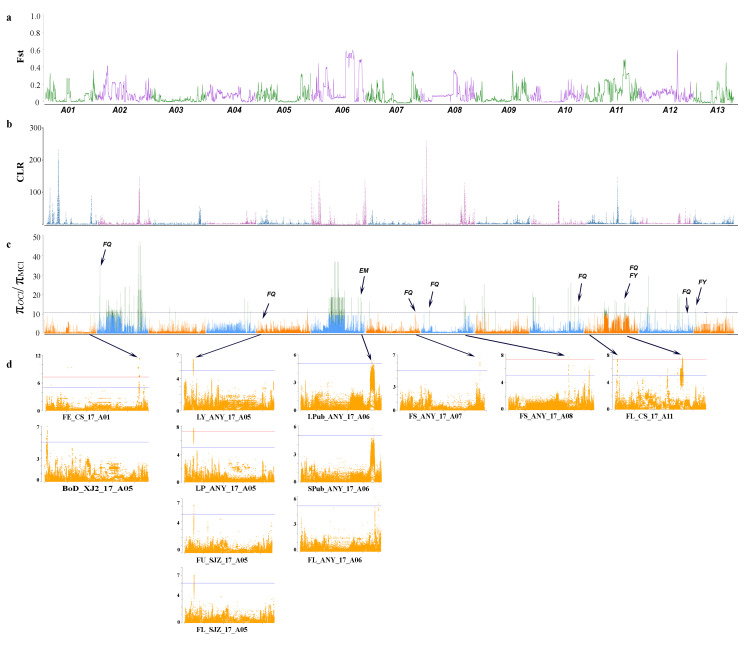
Genetic differentiation and identification of selection sweeps between OCl and MCl on At sub-genome (**a**) Population fixation statistics (*F*st) for At sub-genome (Chr A01–A13). A threshold of top 1% is selected as highly differentiated regions, (**b**) CLR (Composite likelihood ratio) score as an estimate of genome-wide selection sweeps, (**c**) π_OCl_/π_MCl_ values (genetic diversity in the cultivated group as compared to obsolete cultivars from Southwest China) for A genome (Chr A01–A13). π ratio was calculated using whole-genome data with a 100 kb sliding window. The horizontal dotted line represents the threshold of 1% values, whereas the threshold is represented with green columns. The annotations represent as FQ = Fibre quality [28,29,30,31,32], FY = Fibre yield [30] and EM = Early maturity [31,33,34] which donates to previously identified hotspots/quantitative trait loci (QTLs) on the corresponding location, (**d**) Genome-wide association studies’ (GWAS) results as Manhattan plots of multiple traits where purple horizontal line represents suggestive significant threshold with −log10(1 × 10^−5^), and the red horizontal line represents genome-wide significant threshold with −log10(5 × 10^−8^). FE = Fibre elongation, BoD = Boll opening Days, LY = Lint yield, LP = Lint percentage, FU = Fibre length uniformity, FL = Fibre length, LPub = Leaf pubescence, SPub = Stem pubescence, FS = Fibre strength. ANY = Anyang, Henan Province, CS = Changsha, Hunan Province, SJZ = Shijiazhuang, Hebei Province.

**Figure 3:**
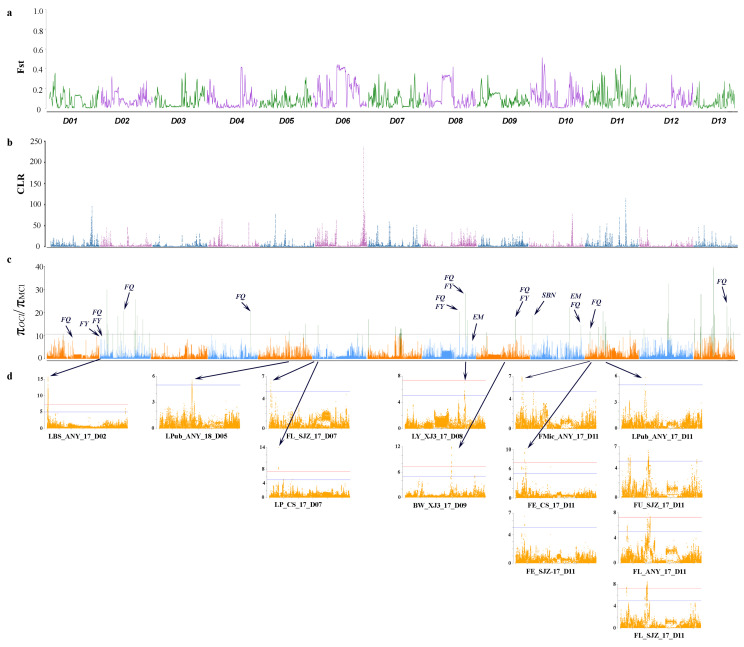
Genetic differentiation and identification of selection sweeps between OCl and MCl on Dt sub-genome (**a**) Population divergence (*F*st) for Dt sub-genome (Chr D01–D13). A threshold of top 1% is selected as highly differentiated regions, (**b**) CLR (Composite likelihood ratio) score as an estimate of genome-wide selection sweeps, (**c**) π_OCl_/π_MCl_ values (genetic diversity in the cultivated group as compare to obsolete cultivars from Southwest China) for D genome (Chr D01–D13). π ratio was calculated using whole-genome data with a 100 kb sliding window. The horizontal dotted line represents the threshold of top 1% values, whereas the threshold is represented with green columns. The annotations represent FQ = Fibre quality [28,29,30,31,32], FY = Fibre yield [30], SBN = Sympodial branch node and EM = Early maturity [31,33,34] which donates to previously identified hotspots/QTLs on the corresponding location, (**d**) GWAS results as Manhattan plots of multiple traits. LBS = Leaf base spot, BW = Boll weight, FMic = Fibre micronair, FE = Fibre elongation, LY = Lint yield, LP = Lint percentage, FU = Fibre length uniformity, FL = Fibre length, LPub = Leaf pubescence. ANY = Anyang, Henan Province, CS = Changsha, Hunan Province, SJZ = Shijiazhuang, Hebei Province, XJ3 = Shihezi, Xinjiang Province.

First, the genetic differentiation between modern cultivars (MCl) and obsolete cultivars (OCl) was estimated (Figure 2a and Figure 3a). Population fixation statistics (*F*st) estimates resulted in understanding the differentiation between two groups of cultivars. Comparatively higher differentiation was associated with chromosomes A06, A08, A09 and A11 on sub-genome At (Figure 1a), while relatively high differentiation was estimated for D-genome on chromosomes D03, D04, D06, D07, D08, D10 and D11. Furthermore, we selected the top 5% threshold to select highly differentiated regions between the two groups of cultivars. With the threshold of *F*st > 0.2975, we identified 193 highly differentiated regions. Among these regions, 103 reside on At sub-genome, and 90 reside on Dt sub-genome (Supplementary Table S3). Selection bottlenecks resulted in the loss of genetic diversity and depletion of elite alleles conferring favourable phenotypes in crop plants. To identify regions potentially associated with selection pressure for improvement, we scanned genomic regions with the highest reduction in diversity in modern cultivars and obsolete accessions. Furthermore, we selected a genome-wide top 1% threshold of reduction of diversity (ROD) π_OCl_/π_MCl_ > 10.425 and categorise these regions as selection signals (Supplementary Table S4). A total of 311 improvement signals were identified, while 235 regions were located on At sub-genome, and the remaining 76 were located on the Dt sub-genome (Supplementary Table S4). The identified selection signals were compared with previously published reports. Some of the signals overlapped with previously reported QTLs for fibre yield and fibre quality. Contrary to previous reports, we found multiple hotspots for selection pressure on chromosome A02, A06, A11 on At sub-genome (Figure 2), D02, D10, D11, D12 and D13 on Dt sub-genome (Figure 3). Besides, we mapped the GWAS results of multiple traits with selection signals (Figure 2c and Figure 3c). 

### 2.3. Differentiation and Domestication between G. hirsutum Landraces and Cultivar Groups

**Figure 4:**
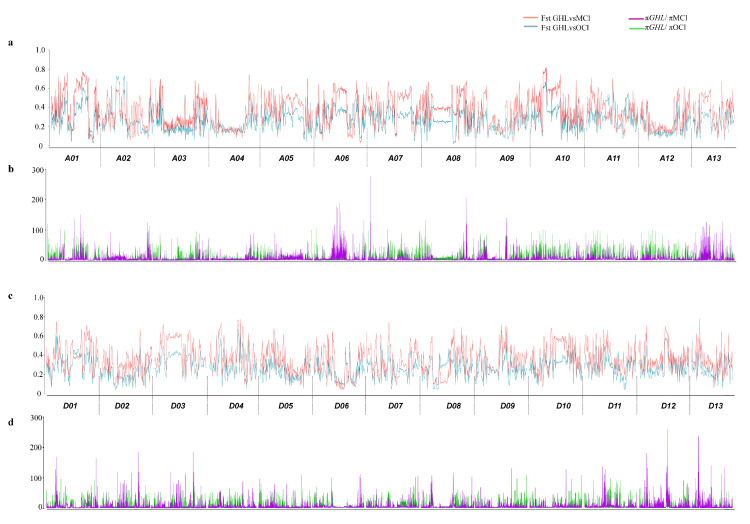
Genetic differentiation and selection signals among *G. hirsutum* landraces (GHL), modern cultivars (MCl) and Obsolete cultivars (OCl) (**a**) Population divergence (*F*st) for At sub-genome (Chr A01–A13), (**b**) π*_GHl_*/π_MCl_ (Purple columns) and π*_GHl_*/π_OCl_ (Green columns) values (genetic diversity in *G. hirsutum* landraces (GHL) as compared to MCl and OCl for At sub-genome (Chr A01–A13), (**c**) Population divergence (*F*st) for Dt sub-genome (Chr D01–D13), (**d**) π*_GHl_*/π_MCl_ (Purple columns) and π*_GHl_*/π_OCl_ (Green columns) values (genetic diversity in *G. hirsutum* landraces (GHL) as compare to MCl and OCl for Dt sub-genome (Chr D01–D13).

To understand the differentiation and domestication between *G. hirsutum* landraces and cultivar groups, we compared the landraces of *G. hirsutum* with modern cultivars and obsolete accessions. Genome-wide population fixation statistics (*F*st) suggested a wide range of genetic differentiation among these groups. *G. hirsutum* landraces showed significantly higher differentiation than modern cultivars and other obsolete accession collected from Southwest China, which is consistent with the breeding history of cotton (Figure 4). At sub-genome showed relatively higher differentiation as compare to Dt sub-genome (Figure 4a,d). Chromosome A03, A04, A12, A08, D05 and D08 showed less differentiation when compared with GHL, suggesting the conserved nature of these regions on the respective chromosomes. Furthermore, chromosomes A01, A02, A05, A06, A07, A10, D03, D04, D09, D10 and D11 depicted higher differentiation, suggesting the accumulation of changes due to adaptation, selection and improvement during the past few decades. We also investigated the diversity ratio to understand the genome-wide selection during domestication from landraces to modern cultivars. A large number of selection signals were identified genome-wide. Modern cultivars (MCl), as compared to Obsolete accessions from Southwest China (OCl) showed higher peaks representing selection sweeps, which is consistent with the breeding history of cotton in China and also emphasises the fact that modern cultivars are the result of rigorous selection over the period of time. Chromosome A06 showed significant selection signals, while the same region on chromosome A06 showed a comparative differentiation among three groups; these results emphasised on change in genomic structure due to selection and improvement. A similar pattern of variation was observed in chromosome A13. Dt sub-genome also showed considerable selection sweep signals genome-wide. A similar pattern of selection sweeps was observed in At sub-genome, where modern cultivars showed higher selection peaks than obsolete cultivars. 

### 2.4. GWAS

Early maturity and improvement in fibre quality have been major objectives of breeding projects during the last century. To identify putative candidate genes for fibre yield, fibre quality and flowering time, we conducted a genome-wide association study (GWAS) using phenotypic data collected in 2017 and 2018. We selected 4,329,838 high-quality SNPs with minor allele frequency >0.05. The high-density map was found to be superior to previous reports [19,27]. A total of 25 association signals were identified with *p* < 4.9 × 10^−7^ by using efficient mixed-model association expedited (EMMAX) (Supplementary Figure S1a–d). Very few of these associations have been previously characterised. We identified significant GWAS signals for fibre yield on chromosomes A05, D06, D08 and D09. These GWAS signals are also associated with significant improvement signals present on the respective chromosomes. We also identified 16 significant GWAS associations for fibre quality traits viz. fibre length (FL), fibre elongation (FE), fibre length uniformity (FU), fibre strength (FS) and fibre micronair (FMic). These signals were present of chromosome A01(FE), A05 (lint percentage (LP), FU, FL), A06 (FL), A07 (FS), A08 (FS), A09 (FL) on At sub-genome, while D06 (FL), D11 (FMic, FE, FU and FL) on Dt sub-genome. These identified signals, key SNPs, and their corresponding annotation have been presented in supplementary Table S5.

## 3. Discussion

The *Gossypium* genus includes 50 species distributed worldwide in tropical and subtropical regions. *Gossypium hirsutum* is the most important species among all due to its high yield and spinnable fibre quality for industrial use [35]. Upland cotton, a type of *G. hirsutum,* dominates worldwide and has been the primary source of fibre production, as it has been growing on more than 90% of cotton-growing areas. Upland cotton originated in Mesoamerica, from where it spread worldwide through trade routes during the 18th century. In the early 20th-century, upland cotton was systematically introduced to cotton-growing countries worldwide, including China, India and Australia, which lead to the development of the modern cotton industry. Since upland cotton was developed from limited resources [36,37], it is considered that subsequent introduction and spread of upland cotton worldwide has reduced its genetic diversity. Reduction in diversity can negatively influence the development of superior crop varieties [38,39]. A comparison of two periods of the introduction of upland cotton in China can provide insight into the process of domestication and improvement in cultivars and changes in genomic structure. Thus, we evaluated three groups of genotypes, including modern cultivars, obsolete accessions collected from Southwest China and geographical landraces of *G. hirsutum*. 

Phylogenetic, principal component and structure analyses indicated the divergent behaviour of modern cultivars with comparison to OCL and GHL, which were in agreement with genetic differentiation analysis. The divergent trends of landraces compared to modern cultivars are also in agreement with previous studies [18,27,38,40,41,42]. In support of previously published works, which have suggested narrowed genetic diversity among studied cultivars of upland cotton and also in other crops [9,27,43], our results also emphasised reduced differentiation on a genetic level corresponding to modern cultivars. Chen et al. [37] reported genetic diversity in source germplasm comprising of 43 upland cotton accession using simple sequence repeats (SSR) markers and concluded a decrease in genetic diversity in modern cultivars. Genetic bottlenecks in crop domestication may have resulted in the loss of genetic diversity and elite alleles in modern cultivars [44,45]. However, wild progenitors and landraces are excellent sources for developing desirable variations in current cultivars [46].

Furthermore, Obsolete accessions collected from southwest China are a rich source of genetic information for comparison of genetic variation in modern cultivars because of domestication and improvement. These genotypes comprised a distinct gene-pool, which is not only from a different ecosystem but also with less systematic selection. A comparison of these accessions with modern cultivars can provide insight into changes in genomic structure due to human-mediated selection. Therefore, we analysed these accessions and compared them with MCl and GHL. Our results suggested a marked differentiation between OCl, MCl and GHL. A comparison of GHL with OCl showed lower differentiation as compare to MCl. The divergent behaviour of geographical landraces of *G. hirsutum* was in accordance with the genetic differentiation analyses. This divergent trend of landraces compared to modern cultivars and obsolete accessions was also found in agreement with previous studies [18,27,38,40,41,42]. Furthermore, selection pressure as improvement/selection signals in obsolete cultivars (OCl) was lower than modern cultivars (MCl). These results are consistent with the breeding history of upland cotton in China [37]. 

Besides, we identified a large number of selection sweeps, suggesting the domestication bottlenecks. Some of the identified selection sweeps overlapped with highly differentiated regions on respective chromosomes, i.e., A06, A08, A09, A10, A11, D02, D04, D10, D12 and D13. This overlapping pattern suggests the occurrence of differentiation due to human-mediated selection. Further, to understand the genetic basis of domestication and improvement in fibre yield, fibre quality, maturity and other morphological traits, we compared the location of selection sweeps with the significant loci of GWAS analysis and narrowed down selection sweeps into corresponding small regions which will be helpful for future studies to determine and characterise new genes concerning domestication and selection in upland cotton. Some of the selection sweeps we identified have been previously reported for fibre yield [30], fibre quality [28,29,30,31,32] and maturity [31,33,34]. Fang et al. [18] performed a comprehensive experiment for identification of selection signature in 318 upland cotton accession and consequently identified 15 regions associated with improvement through comparison of whole-genome diversity between modern cultivars and landraces. However, with improved sequencing technology, our study resulted in a better understanding of selection/improvement signals. Improvement signals (π_OCl_/π_MCl_) were lower than selection signals (π*_GHl_*/π_MCl_), suggesting a weaker selection pattern during modern genetic improvement than earlier selection [47,48]. These results can lead us to understand the changes at the genomic level caused by domestication, selection and improvement of upland cotton cultivars. Modern sequencing technology and GWAS has enabled us to better understand the genetic mechanisms behind the evolution of a specific trait, as previously described in different crops [40,49,50]. In this study, we identified multiple GWAS signals significantly associated with different traits, including fibre yield and quality. Moreover, loci associated with fibre yield (chromosome A05, D06 and D08), fibre quality (chromosome A01, A05, A07, A08, A11, D01, D07, D08 and D11) and other morphological traits (chromosome A01, A06, D02, D05 and D11) fall within the selection sweeps, and these loci have not been previously reported. Genotyping for these traits and identification of candidate genes and their functional analysis can reveal the potential impact of genes related to traits.

## 4. Materials and Methods 

### 4.1. Plant Material 

A total of 357 upland cotton accessions obtained from the gene bank of the Cotton Research Institute of the Chinese Academy of Agricultural Sciences (CRI-CAAS) with diverse genetic backgrounds were used for phenotyping. These accessions comprised three groups, i.e., group 1 belonged to modern cultivars (235) currently being cultivated, group two comprised 91 obsolete cultivars collected from southwest China and group 3 (31) comprised seven reported geographical landraces of *G. hirsutum,* i.e., Yucatanese, richmondi, morrilli, Marie-Galante, palmeri, punctatum and latifolium (Group 3 was not included in phenotyping as these landraces cannot flower in the test locations due to photoperiod sensitivity) (Supplementary Table S1). Two replications were planted in five agro-ecologically different environments viz. Shijiazhuang (SJZ) in Hebei Province, Changsha (CS) in Hunan province, Anyang (AY) in Henan Province (Yellow River region), Alaer and Shihezi in the Xinjiang (XJ) autonomous region (Northwest Inland), for two consecutive seasons 2017 and 2018. Two sets of genotypes were used for phenotyping Set 1 comprised 169 accessions whose phenotypic data was collected from SJZ, CS, AY, XJ2 and XJ3, Set 2 comprised 324 accessions whose phenotypic data was collected from AY. Some of the genotypes in both sets overlapped to give a proper representation of two groups viz. G1-MCl = Modern cultivars G2-OCl = Obsolete Cultivars. All standard field management practices were applied, including irrigation, pest management and fertilisation, following the usual local management practices in each test location. The cotton was sown in mid- to late-April and was harvested in mid- to late-October at all locations.

In all test locations, phenotypic traits were recorded following the same scoring standard. We characterised lint yield (LY), lint percentage (LP), fibre quality (fibre length (FL, mm), fibre length uniformity (FLU, %), fibre micronair (FMic), fibre strength (FS), fibre elongation (FE, %)), flowering time (DF, days), boll opening days (BoD, days), leaf pubescence (LPub) and stem pubescence (SPub). Fibre quality was tested using twenty naturally opened balls from each accession. A High-volume instrument (HFT9000) was used for characterizing fibre quality parameters at the Cotton Quality Testing Center in Anyang, China. Flowering time was observed daily, and days to flowering (DF) were calculated from the sowing day to the day that the first flowers appeared in 50% of the plants. All samples were subjected to the High-volume instrument (HFT9000) for the estimation of quality parameters. 

### 4.2. DNA Extraction, Sequencing, Alignment and SNP Detection 

Total genomic DNA was extracted from the seedlings of five cultured seeds of each accession in a growth chamber. After three weeks of sowing, at the true leaf stage, young leaves were collected, and a Plant DNA Mini Kit (Aidlab Biotech, Beijing, China) was used to extract total genomic DNA. Three hundred and fifty base pair whole-genome libraries were constructed for each accession according to the manufacturer’s specifications (Novogene Bioinformatics technology company, Beijing, China). Subsequently, we used Illumina HiSeq X10 by a commercial service “Novogene” platform to generate 6.45-Tb raw sequences with 150-bp read length. Following alignment of high-quality reads with the genome of *G. hirsutum*, GATK (Genome Analysis Toolkit, version v3.1) was used for SNP calling. Sequencing data for G4; GHL was obtained from published data [18].

### 4.3. Population Genetic Analyses

Population structure was studied using ADMIXTURE [51], which utilises a clustering method (mode-based) to draw population structure assuming different numbers of clusters (K). A total of 431,985 SNPs without missing genotypes were used. SNPhylo software was used to prune SNPs, which reduces SNP redundancy by linkage disequilibrium (LD). SNPs in the same LD block provide redundant lineage information. SNPhylo keeps only one informative SNP in a LD block, and subsequently, a relatively small number of SNPs (9.97%) were used for structure and phylogenetic analysis.

Principal component analysis was performed using the EIGENSOFT package with an embedded SMARTPCA program [52] using 4,329,838 SNPs without missing genotypes. 

Phylogenetic analysis was performed to understand phylogenetic relationships among genotypes by constructing a phylogenetic tree using SNPs of all genotypes. SNPs were filtered with minor allele frequency, MAF = 0.05. Subsequently, a neighbour-joining tree was constructed using the maximum likelihood method with SNPhylo software [53]. To visualise the phylogenetic tree, we used Dendroscope.

### 4.4. Identification of Selection/Improvement Signals 

The fixation index (*F*st) is a measure of population differentiation as it provides insight into the genome-wide differentiation among different groups. Thus, we calculated population fixation statistics (*F*st) using vcftools with a sliding window of 100 kb and step size of 20 kb (--fst-window-size 100,000 --fst-window-step 20,000). The average *F*st of all sliding windows was considered as the value at the whole genome level among different groups. 

Highly diverged regions were selected by merging fragments with a distance of less than 50 kb after the initial selection of the top 1% π values. To identify the putative regions under selective pressure between landraces and cultivars, the nonsynonymous SNPs with the top 1% of *F*st values were selected.

Nucleotide diversity (π) is an estimate of the degree of variability within population and species [54]. Nucleotide diversity was calculated using vcftools with a 100 kb sliding window based on genotypes in different groups separately. Furthermore, genetic diversity ratios between different groups were calculated to estimate selection/improvement regions. π_OCl_/π_MCl_ was used as an estimate of improvement signals, while π_GHL_/π_MCl_ and π_GHL_/π_OCl_ were used as an estimate of selection signals. The top 1% threshold was used to identify significant selection/improvement signals. Composite likelihood ratio (CLR) was calculated as an alternative estimate for selection/improvement signals, using SweeD. Diversity ratios and CLR scores were compared for better assessment of selection/improvement signals. 

### 4.5. GWAS Analysis 

For GWAS analysis, we categorised genotypes into two sets. Set 1 comprised 169 accessions whose phenotypic data were collected from SJZ, CS, AY, XJ2 and XJ3, Set 2 comprised 324 accessions whose phenotypic data were collected from AY. A total of 4,329,838 high-quality SNPs were subjected to filtering with MAF >0.05, missing rate <20% and 1,604,221, and 1,506,091 SNPs were kept in set 1 and set 2, respectively, and subsequently, GWAS was performed on both sets of genotypes separately. Accessions with missing SNPs data were excluded from analyses. We performed GWAS for multiple traits in efficient mixed-model association expedited (EMMAX) software [55,56]. Population stratification and hidden relatedness were modelled with a kinship (K) matrix in the emmax-kin-intel package of EMMAX. The significant threshold for GWAS was kept constant with the suggestive significant threshold at −log10(1 × 10^−5^) and the genome-wide significant threshold at −log10(5 × 10^−8^).

## 5. Conclusions

Our study provides a genetic basis to understand the domestication process in upland cotton cultivars. It also provides a comprehensive insight into changes in genome structure due to selection and improvement during the last century. We also identified multiple GWAS associations for fibre yield, quality and other morphological characteristics. Further study is required to explore these novel loci associated with different traits to uncover causal genes related to these traits. Our study provides a comprehensive insight into the differentiation between modern cultivars, OCl and GHL, which can be a useful tool for the cotton breeders to understand changes accumulated due to selection and improvement breeding strategies.

## Supplementary Materials

The following are available online at https://www.mdpi.com/2223-7747/9/6/711/s1, Figure S1 (a) Manhattan plots for GWAS results, (b) Manhattan plots for GWAS results, (c) Manhattan plots for GWAS results, (d) Manhattan plots for GWAS results; Table S1 list of accessions, cultivars and landraces used in this study; Table S2 Structure results and corresponding grouping for all accessions and landraces used in this study; Table S3 List of highly differentiated regions and genes located in the highly differentiated regions identified through pairwise *F*st estimates (top 1% threshold); Table S4 Improvement signals, Top 1% threshold of πOCl/πMCl; Table S5 Key SNPs identified for flowering time, fibre quality and anther colour, and their corresponding annotations.
