# Supplementary material for: Genomic Insight into Differentiation and Selection Sweeps in the Improvement of Upland Cotton
Source: Plants (Basel). 2020 Jun 3;9(6):711. doi: 10.3390/plants9060711 (PMC7356552; doi:10.3390/plants9060711)
Supplement: Supplementary file 1 [file plants-09-00711-s001.zip › Supplementary Figures.docx]

**Supplementary Figures**


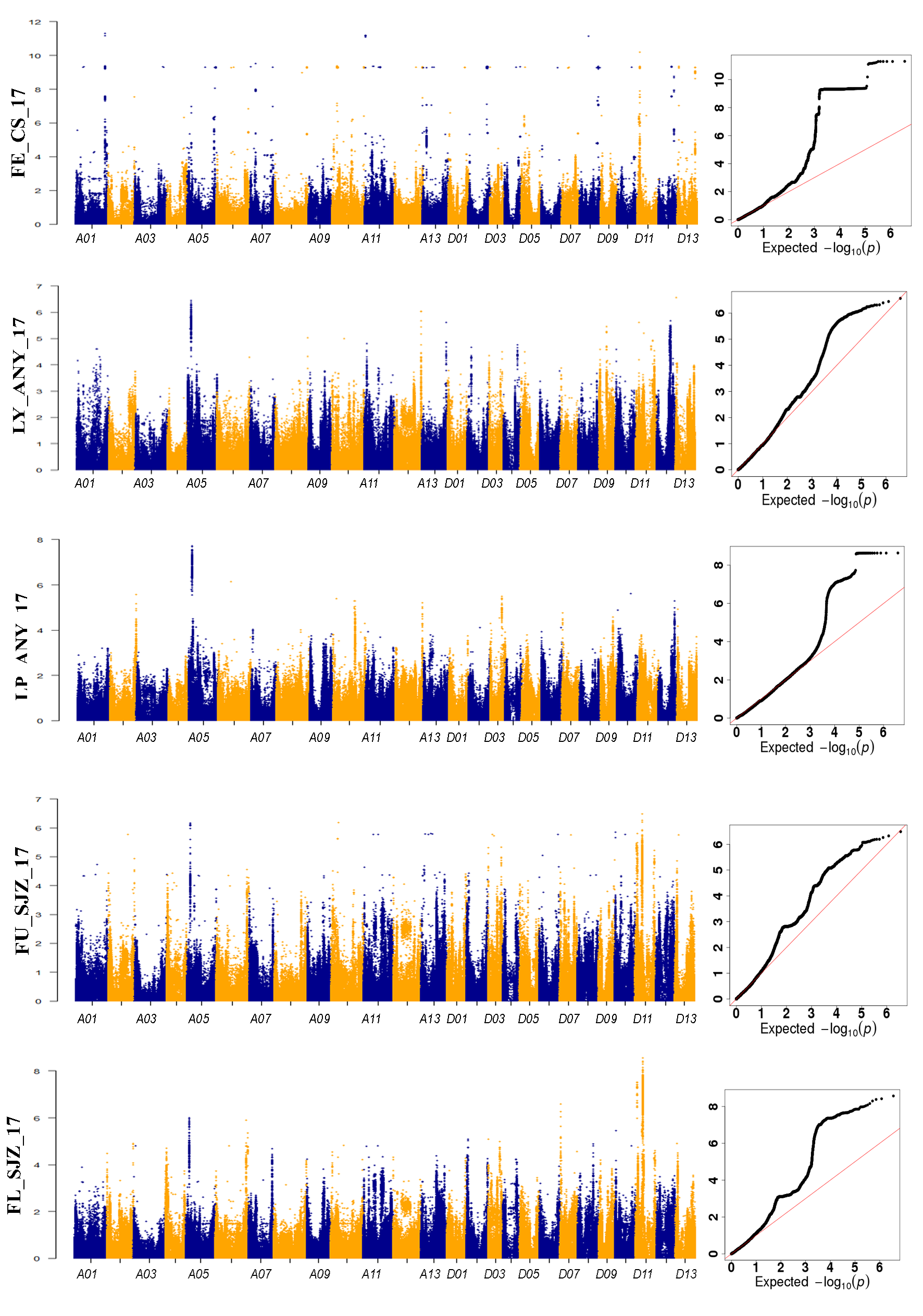


**Figure S1a**. Manhattan plots and QQ plots for GWAS results corresponding to multiple traits

*FE=fiber elongation, LY= Lint yield, LP= Lint percentage, FU= Fiber length uniformity, FL= Fiber length

# ANY= Anyang, Henan province, CS=Changsha, Hunan province, SJZ= Shijiazhuang, Hebei Province


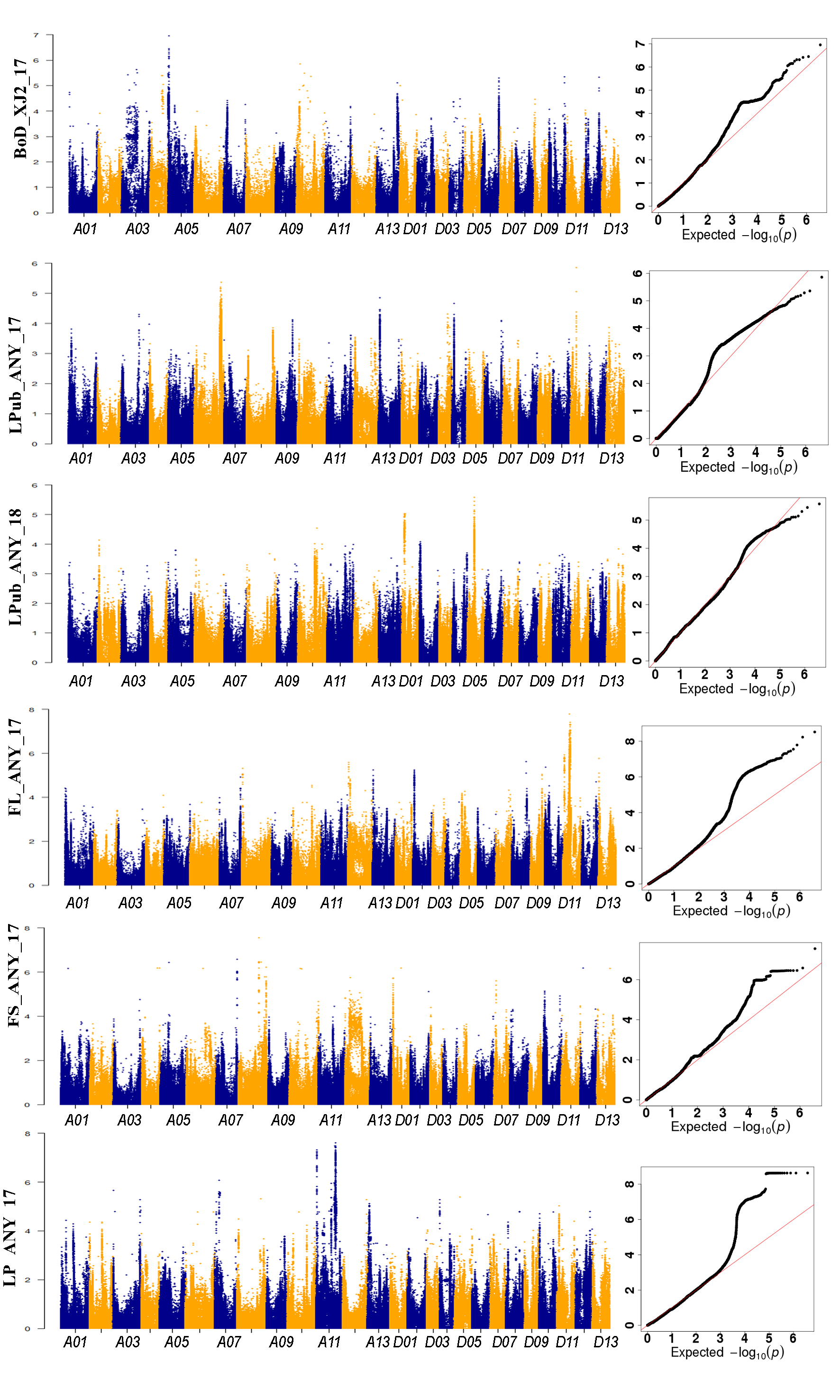


**Figure S1b**. Manhattan plots and QQ plots for GWAS results corresponding to multiple traits

*BoD= days to boll opening, LPub= Leaf pubescence, LP= Lint percentage, FS= Fiber strength, FL= Fiber length

#ANY= Anyang, Henan province, XJ2= Alaer, XinJiang province


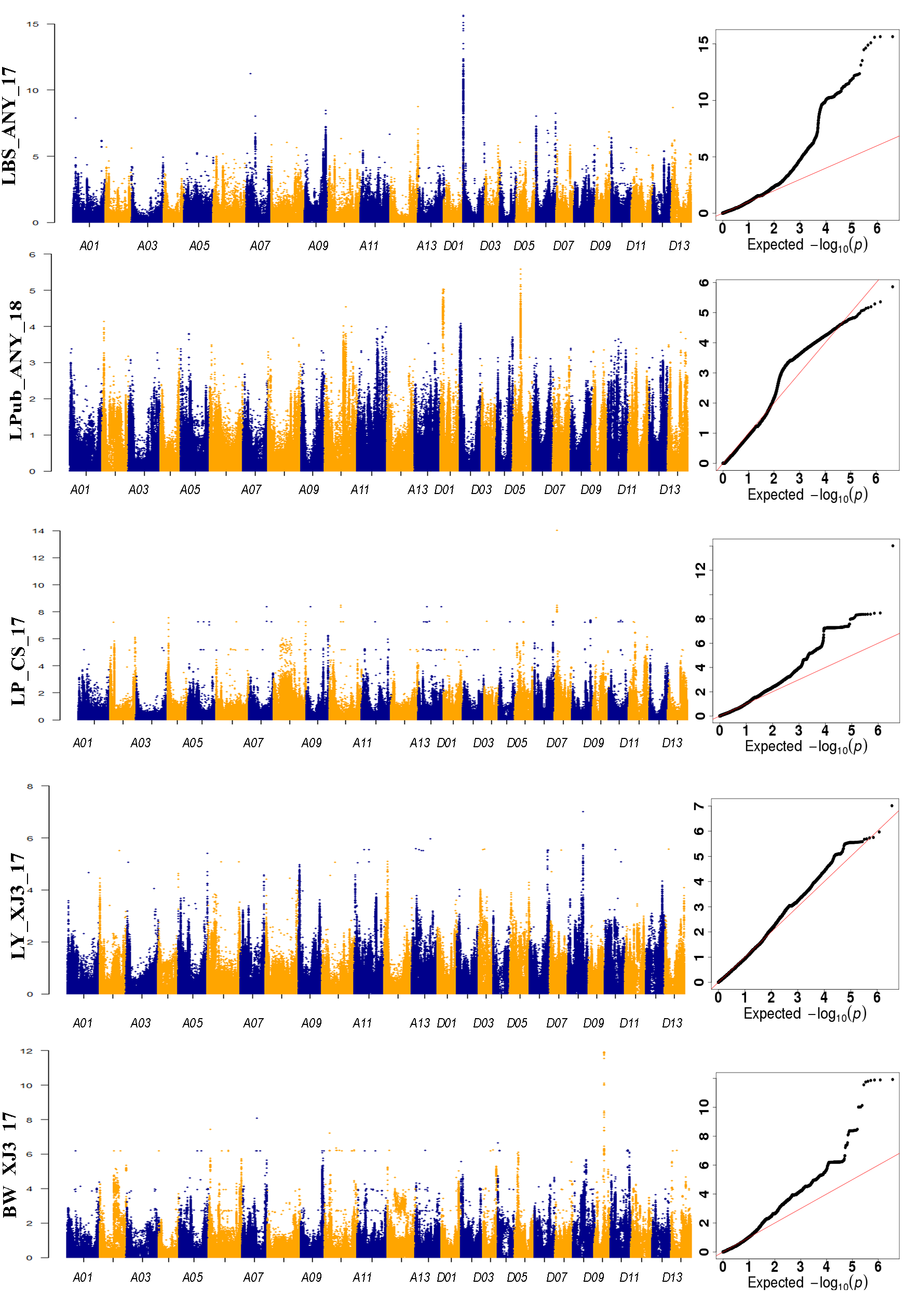


**Figure S1c**. Manhattan plots for GWAS results

*LBS= Leaf base spot, LPub= Leaf pubescence, LP= Lint percentage, LY= Lint yield, BW= Boll weight

# ANY= Anyang, Henan province, CS=Changsha, Hunan province, XJ3= Shihezi, XinJiang province,


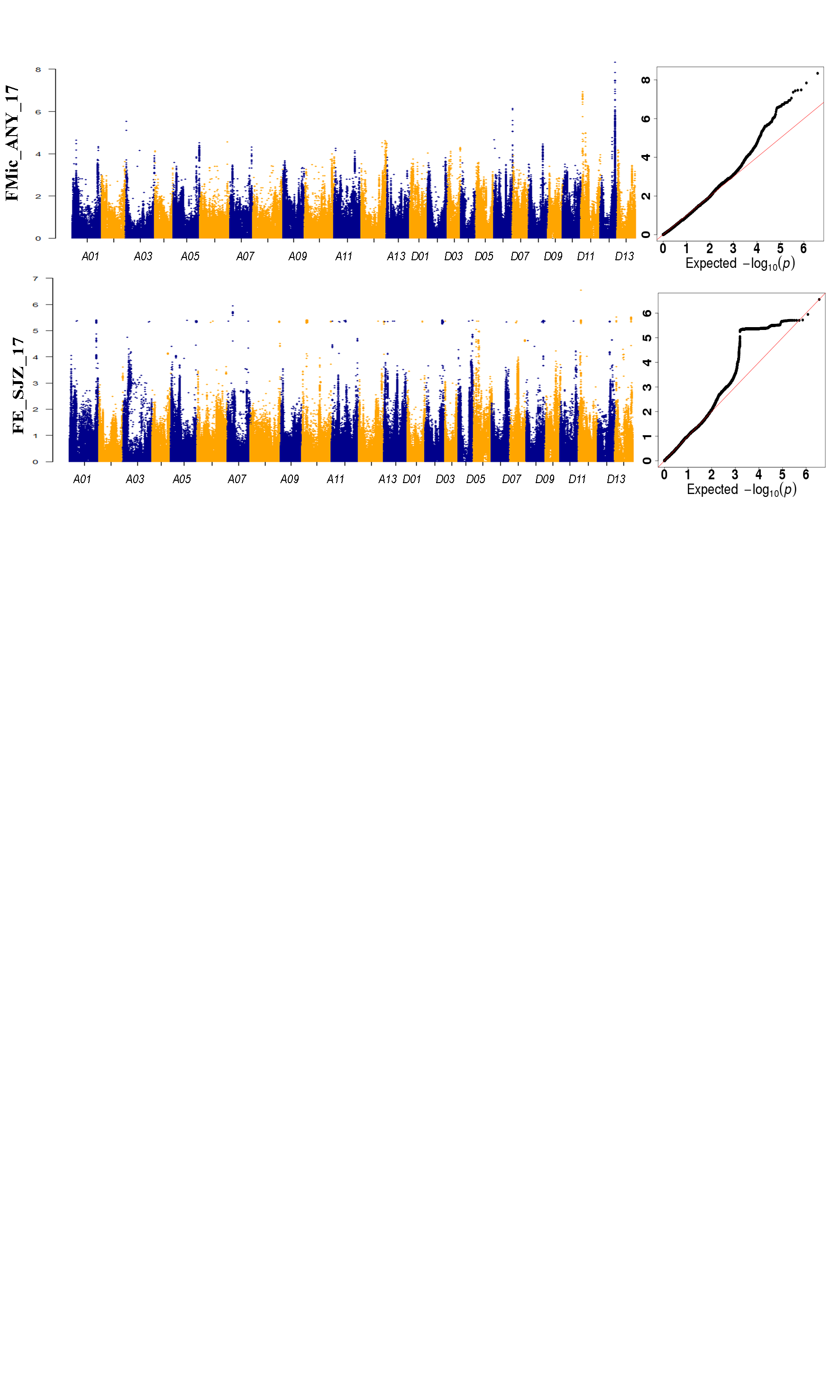


**Figure S1d**. Manhattan plots and QQ plots for GWAS results corresponding to multiple traits

*FMic= Micronair, FE= Fiber elongation

# ANY= Anyang, Henan province, SJZ= Shijiazhuang, Hebei Province
